# NextClip: an analysis and read preparation tool for Nextera Long Mate Pair libraries

**DOI:** 10.1093/bioinformatics/btt702

**Published:** 2013-12-02

**Authors:** Richard M. Leggett, Bernardo J. Clavijo, Leah Clissold, Matthew D. Clark, Mario Caccamo

**Affiliations:** The Genome Analysis Centre (TGAC), Norwich Research Park, Norwich NR4 7UH, UK

## Abstract

**Summary:** Illumina’s recently released Nextera Long Mate Pair (LMP) kit enables production of jumping libraries of up to 12 kb. The LMP libraries are an invaluable resource for carrying out complex assemblies and other downstream bioinformatics analyses such as the characterization of structural variants. However, LMP libraries are intrinsically noisy and to maximize their value, post-sequencing data analysis is required. Standardizing laboratory protocols and the selection of sequenced reads for downstream analysis are non-trivial tasks. NextClip is a tool for analyzing reads from LMP libraries, generating a comprehensive quality report and extracting good quality trimmed and deduplicated reads.

**Availability and implementation:** Source code, user guide and example data are available from https://github.com/richardmleggett/nextclip/.

**Contact:** Richard.Leggett@tgac.ac.uk

**Supplementary information:** Supplementary data are available at *Bioinformatics* online.

## 1 INTRODUCTION

**Fig. 1.**
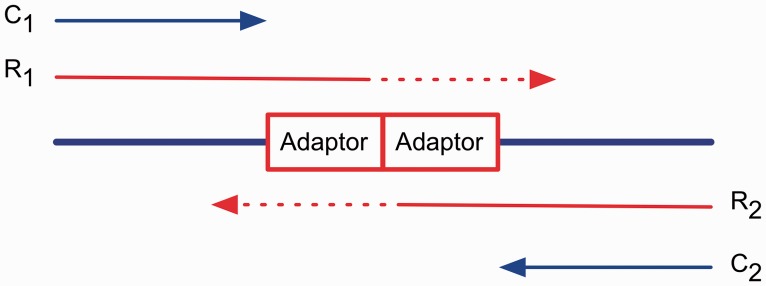
Nextera mate pair fragments are formed by the joining of two junction adaptors. Reads R_1_ and R_2_ are produced from both ends and are clipped at the adaptor to produce C_1_ and C_2_

Long Mate Pair (LMP) reads are an important tool in the scaffolding of complex genome assemblies because they allow bridging of large repeat regions. Equally, long-range information provided by LMP libraries is one of the key tools used for the characterization of structural variants. However, LMP libraries can be technically challenging to make requiring large amounts of high-quality and high-molecular weight DNA and generating low library yields with variable levels of contaminants that are best removed before scaffolding. Illumina’s recently released Nextera mate pair sample preparation kit (Illumina FC-132-1001) is an attractive system providing library insert sizes of up to 12 kb, while requiring less DNA and generating high-complexity libraries (Park *et al.,* 2013). Under the Nextera protocol, a transposase enzyme fragments DNA and attaches a 19 bp biotinylated adaptor to either end of each fragment in a process known as ‘tagmentation’. The ‘tagmented’ DNA is circularized, resulting in the joining of the two biotinylated junction adaptors. The circularized DNA is fragmented and biotin enrichment used to obtain the fragments containing the adaptors that mark the junction. During sequencing, reads are produced from both ends of a fragment, reading inwards toward and through the junction adaptors (Fig. 1).

In an ideal library, the junction adaptor would appear in the middle of every fragment and the fragments would be sized such that the adaptor is found in the last 19 bases of each read, resulting in most of the read being available for use. In reality, the adaptor can occur anywhere in the read and the read has to be trimmed at the point the adaptor is found (Illumina, 2012). Similarly, fragments can be large enough that the adaptor does not appear in either of a pair of reads. A related problem is that the biotin enrichment process is imperfect, meaning that some paired-end fragments not containing junction adaptors are also sequenced. These fragments are impossible to tell apart from fragments that contain the adaptor, but are too long for the adaptor to be sequenced. As well as the complexities associated with presence and positioning of adaptors, for a mate pair library to be useful for scaffolding, it needs to have a reasonably tight distribution of insert sizes and a low number of polymerase chain reaction (PCR) duplicates, chimeric inserts and paired-end contaminants. Our own experience, also reported in other work (Park *et al.,* 2013), has established the importance of implementing the right laboratory protocol to produce good quality mate pair libraries. However, quality control of the libraries can require significant bioinformatics analysis. Having produced a suitable library, further processing is required to extract true mate pair reads, remove junction adaptors and clip reads. For this reason we developed NextClip, a tool for comprehensive quality analysis of Nextera LMP libraries and preparation of reads for scaffolding.

## 2 DESCRIPTION OF TOOL

The NextClip package comprises two parts. The core component is the NextClip command line tool, an efficient C program for processing mate pair FASTQ files, generating summary statistics and preparing reads for use in scaffolding. A second component, the NextClip pipeline, is designed for use in cases where there is a partially complete assembly (e.g. contigs from paired-end data) or a close reference. It uses the NextClip tool, along with the alignment tool BWA (Li and Durbin, 2009) to generate a more detailed report that includes analysis of library insert sizes. 

### 2.1 The NextClip tool

NextClip proceeds by examining each pair of reads in a given set of FASTQ files and looking for the presence of the junction adaptor. The program options allow the user to specify how strict a match is required for this stage, but the default is to look for 18 of the 19 junction adaptor bases, or for 34 of the 38 bases from a pair of adaptors (one forward, one reverse compliment). Pairs of reads are classified into one of four categories:

- Category A pairs contain the adaptor in both reads.
- Category B pairs contain the adaptor in only read 2.
- Category C pairs contain the adaptor in only read 1.
- Category D pairs do not contain the adaptor in either read.

NextClip will separate the input FASTQ files into separate files representing each category, with reads trimmed up to adaptor starting point. Reads will only be written if the length of the trimmed read exceeds a user-configurable minimum read length (default 25 bp). NextClip will report the percentage of reads in each category and the percentage of reads exceeding the minimum length. This separation of reads is important because for scaffolding a user would typically only use reads from categories A, B and C. Pairs for which no junction adaptor is found are less likely to be true mate pairs and may well be pair end sequences that have slipped through the biotin enrichment process. Pairs where the adaptor is found in only one of the reads could still contain a degenerated version of the adaptor in the other read. To facilitate clipping of these, an option instructs NextClip to reexamine the non-matching read with looser matching criteria, clipping as necessary and moving into a new category E. Another option will always clip a specified number of bases from the end of any read without an adaptor match. This ensures adaptors at the end of reads are clipped where there is insufficient length to trigger a match.

The rate of PCR duplication is another indicator of library quality. With size selection being an important but complexity bottlenecking step in the gel-based version of the Nextera protocol, the amplification steps performed later on are prone to create too many duplicated molecules. NextClip uses a k-mer-based approach to estimate the PCR duplication rate while reads are examined. It does this by using the first 11 bp and middle 11 bp of each read to generate a signature 44-mer. This is stored in a hash table, and if any subsequent read is found to have the same signature, it is marked as a duplicate. Duplicate numbers are reported and, optionally, pairs can be deduplicated from the output files.

Research has highlighted GC biases with earlier Nextera libraries (Marine *et al.*, 2011; Quail *et al.*, 2012), so NextClip has been designed to calculate the overall GC content of a run, as well as outputting the GC profile distribution of the reads.

### 2.2 The NextClip pipeline

The pipeline (Supplementary Fig. S1) begins by running NextClip, followed by alignment of the output files for the four categories using BWA. Alignment is carried out in single-ended mode, and a Perl script parses the resultant SAM files. For each pair of reads, the script identifies whether the reads are in paired-end orientation, mate pair orientation or tandem orientation and calculates the associated insert size. For each category of read pair, the pipeline will produce mate pair, paired-end and tandem insert size histograms. A final two or three page report is output as a LaTeX file, which is then converted to a PDF (Supplementary Fig. S2).

Once reports have been generated, it is easy to compare one library with another and to pick out unusual biases. We have found it particularly useful to compare the numbers of reads in each category, to look at the proportion of reads in mate pair orientation to those in paired end, to understand the tightness of insert size distributions and to look for unusual numbers of small fragments.

The pipeline has been designed to work either in series on a single computer or in parallel on an High Performance Computing system running the LSF or PBS job schedulers. Other schedulers can be used with minimal change. 

## 3 EXAMPLE RESULTS

To demonstrate the downstream improvements possible with NextClip, we sequenced a 251 bp Nextera LMP library of *Arabidopsis thaliana* Col-0 with 5 kb insert size (deposited as ENA accession ERA264981) and assembled reads from this and an already published 100 bp Illumina HiSeq paired-end library (ENA run SRR519624) using ABySS (Simpson *et al.*, 2009). Scaffolding with unprocessed LMP reads resulted in a decrease in the scaffold N50 due to the misleading information contained in unclipped reads and the presence of reads from fragments with no junction adaptor. Processing with NextClip, using categories A, B and C resulted in substantial improvements to scaffold N50 (Table 1).

**Table 1.** ABySS *A.thaliana* assembly with and without NextClip clipping

Reads used for assembly	Contig N50	Scaffold N50
Paired end only	15 627	21 939
PE and all raw LMP	15 627	15 628
PE and NextClip processed A, B and C categories	15 627	245 226

## 4 SUMMARY

NextClip provides the ability to generate a simple easy to understand report that enables at-a-glance appreciation of library quality and simple separation of reads suitable for scaffolding. We have found it an invaluable tool for enabling us to optimize laboratory protocols to get the most out of a valuable library preparation technique.

## Supplementary Material

Supplementary Data
