# Supplementary material for: NextClip: an analysis and read preparation tool for Nextera Long Mate Pair libraries
Source: Bioinformatics. 2013 Dec 2;30(4):566–8. doi: 10.1093/bioinformatics/btt702 (PMC3928519; doi:10.1093/bioinformatics/btt702)
Supplement: Supplementary Data [file supp_30_4_566__index.html]

NextClip: an analysis and read preparation tool for Nextera long mate pair libraries — NextClip: an analysis and read preparation tool for Nextera Long Mate Pair libraries — NextClip: an analysis and read preparation tool for Nextera Long Mate Pair libraries — Supplementary Data 

# NextClip: an analysis and read preparation tool for Nextera Long Mate Pair libraries

## Supplementary Data

files

**Files in this Data Supplement:**

- Supplementary Data - pdf file
