# Supplementary material for: NextClip: an analysis and read preparation tool for Nextera Long Mate Pair libraries
Source: Bioinformatics. 2013 Dec 2;30(4):566–8. doi: 10.1093/bioinformatics/btt702 (PMC3928519; doi:10.1093/bioinformatics/btt702)

Supplementary Information

Supplementary Figure 1: Data flow through the NextClip pipeline. The NextClip tool runs first, generating four pairs of read files for each of the read categories (adaptor in both reads, adaptor in R1 only, adaptor in R2 only and adaptor in neither read). These are then aligned separately with BWA and results parsed with a custom Perl script. Graphs are plotted using R and a final Perl program brings results together and generates a LaTeX file which is converted to a PDF.

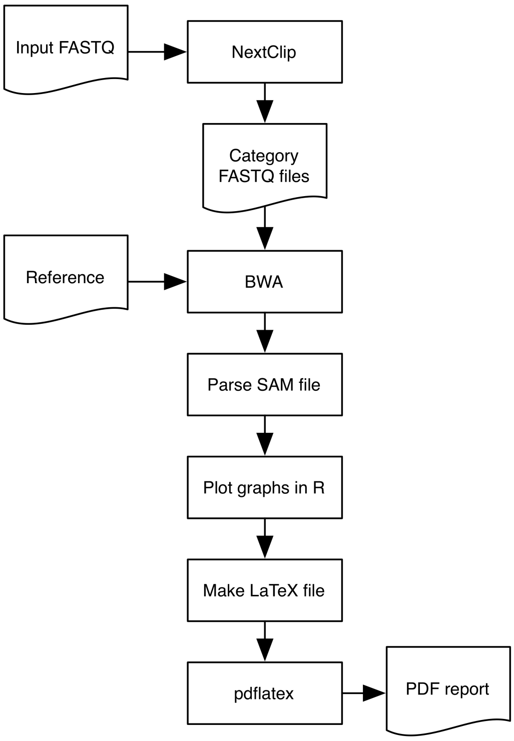

Supplementary Figure 2: Example 3-page NextClip pipeline PDF report.

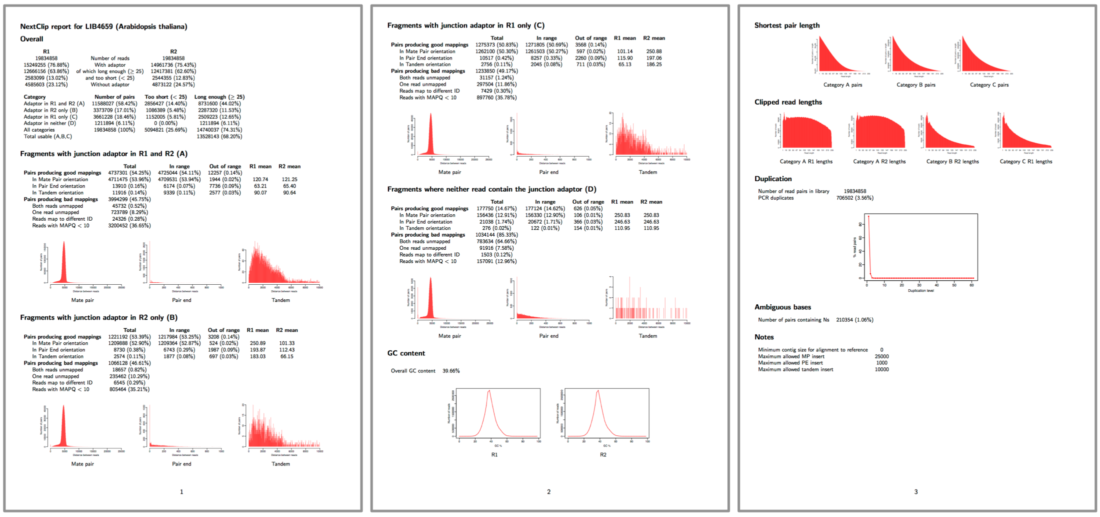

Supplement: Supplementary Data [file supp_btt702_Supplementary.pdf]
